# Large-eddy simulation of vortex-excited force on a square cylinder with transverse wind fluctuations

**DOI:** 10.1038/s41598-023-41470-1

**Published:** 2023-08-30

**Authors:** Huanhuan Du, Zikai Fan, Wei He, Zheguang Yang

**Affiliations:** 1 Wenzhou Key Laboratory of Intelligent Lifeline Protection and Emergency Technology for Resilient City, College of Architecture and Energy Engineering, Wenzhou University of Technology, Wenzhou, 325035 China; 2 Hohai-Lille College, Hohai University, Nanjing, 210000 China; 3 China United Engineering Corporation Limited, Hangzhou, 310051 China

**Keywords:** Civil engineering, Natural hazards

## Abstract

This study utilized 3D Large-eddy simulation to investigate the crosswind drag force on a square cylinder subjected to transverse wind fluctuation. Two distinct methods were employed to generate the fluctuation: a prescribed sine function at the inlet boundary and an upwind barrier. The frequency was normalized in the same Strouhal number form. The transverse wind fluctuation with a normalized frequency above 0.05 tends to excite the square cylinder transversely with the same frequency band. The frequency effect also exists on the square cylinder located downwind an obstacle half the square cylinder’s size. However, an obstacle 2.5 times the size of the square cylinder generates a cross-wind fluctuation with the normalized frequency of 0.04, which cannot excite the square cylinder transversely. The frequency effect from the upwind barrier significantly dampens with the distance and disappears at 8–10 times the square cylinder size.

## Introduction

As the use of light materials in architecture continues to grow alongside economic and technological development, the issue of mitigating cross-wind response in high-rise buildings has become increasingly important for safety and livability^1^. When strong winds approach the building, turbulence, and wake vortices can cause wind-induced loading in the transverse direction of tall buildings^2–5^. As the stiffness of the building decreases, the vortex shedding frequency characterized by the Strouhal number (St) can approach the inherent frequency of the building structure. As wind velocity increases, the vibration frequency of the structure follows the vortex shedding frequency. Once frequency locking occurs, the vibration frequency locks into the natural frequency, with the amplitude of motion increasing significantly compared to the non-locking state^6,7^. It is, therefore, crucial to investigate the dynamic response of buildings in the transverse direction under wind actions and predict the conditions leading to the lock-in state, thus ensuring the safe operation of high-rise buildings^6–11^.

China has been driving the Manhattanization trend, with 51 of the world’s top 100 skyscrapers completed nationwide, with 6 of the top 10 tallest skyscrapers located there, according to the CTBUH Global Tall Buildings Database^12^. This centralization of high-rise buildings changes the wind loading fluctuations experienced by the structure. Additionally, the effect of close-range obstacles makes it challenging to predict vortex-excited forces acting on high-rise buildings. This phenomenon is also observed in box girder structures of long-span bridges, with wind velocity fluctuations failing to adhere to normal distribution even on larger scales^13,14^. The correlation between high-rise buildings’ vortex-induced vibration (VIV) and wind fluctuations on the structure surface is insufficient. The fluctuation in the wind field can cause pressure fluctuations on the building surface with time, making wind vibration response under fluctuating wind fields a critical scientific issue^15^.

Furthermore, most high-rise buildings tend to be in urban built-up areas, such as central business districts (CBDs). Due to the high variability of the underlying surface in urban areas, momentum interaction between airflow and buildings results in complex spatial and temporal turbulent characteristics. It is challenging to obtain reliable VIV predictions based on limited measured data or the mean logarithmic wind velocity profile as the inlet boundary conditions^9^. Therefore, it is essential to develop a deeper understanding of the impact of wind field fluctuations on high-rise buildings, especially within urban environments.

In this study, we conducted large-eddy simulations (LES) to investigate the vortex-excited force (VEF) of a square cylinder under transverse wind fluctuations. The transverse wind fluctuations are generated in two ways: a periodic fluctuating transverse wind velocity utilized by a sine function and a barrier installed upwind. The frequency of vortex-excited force on the square cylinder is analyzed, and the influence of the cross-wind fluctuation is discussed. This study provides insights into the cross-wind drag force of a square cylinder under fluctuating wind fields.

## Method

### Numerical details

In previous reports, the LES model was applied to simulate turbulent flow around bluff bodies^16,17^. Compared to the Reynold-Averaged Navier–Stokes (RANS) approach, the LES method is more accurate in restoring the vortex in a turbulent flow. To explicitly simulate the motion on the dissipative length scale, scientists used the Navier–Stokes equations to model the energy cascade^18,19^. Additionally, a top-hat filter of Smagorinsky was introduced to separate the flow field from the small-eddy field^20^. The LES method was proved applicable in simulating the complex vortex-induced vibration of bridges in wind engineering^21^.

**Figure 1:**
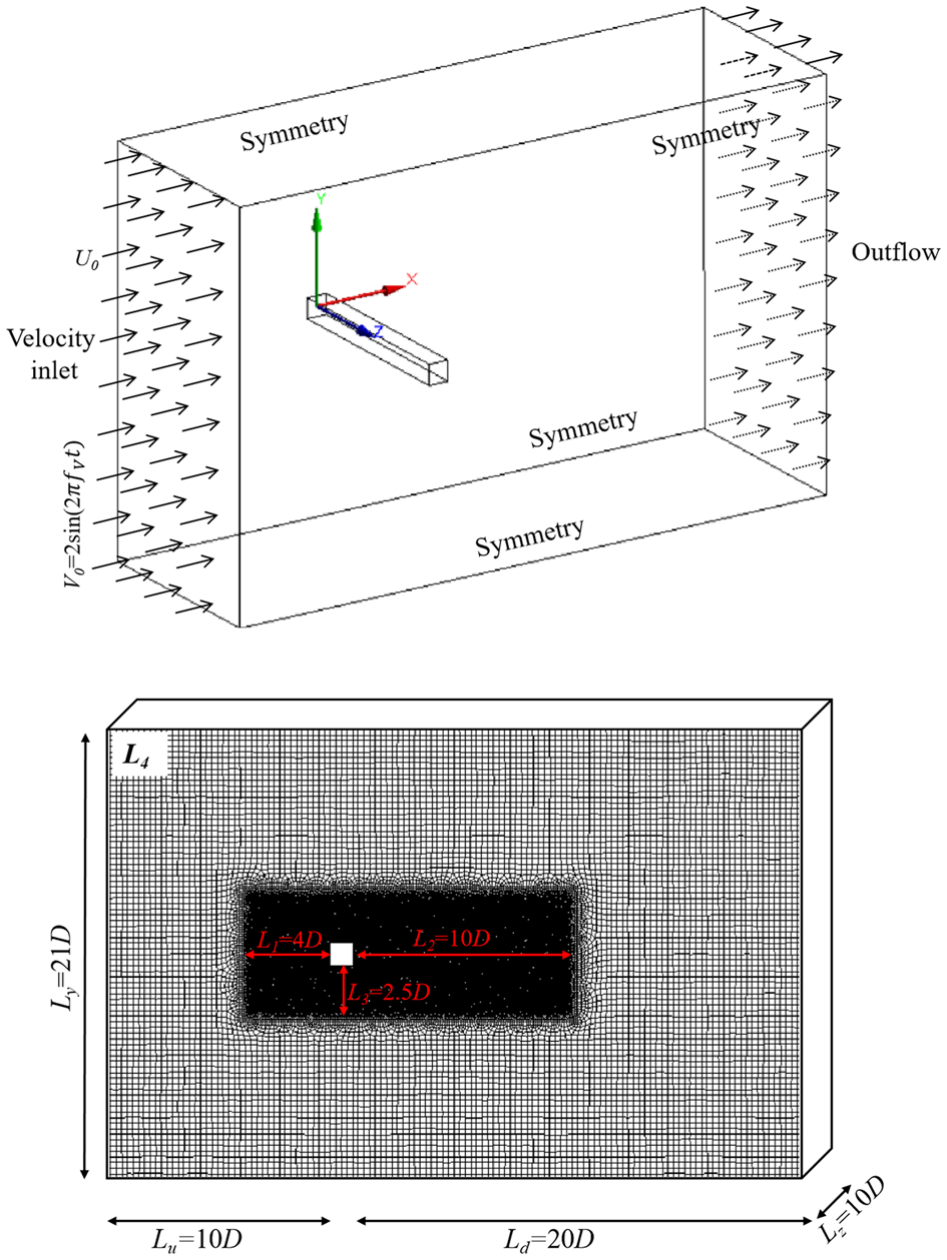
A 3D view of the boundary conditions of the computational domain. A non-uniform mesh system with 1.82 million grids is used.

Figure 1 presents the computational domain around a 3D square cylinder. A square cylinder of size *D* = 0.2 m is exposed to free flow. All geometrical lengths are scaled by *D*. The size of the computational domain is denoted by the normalized length scales *L*_*u*_, *L*_*d*_, *L*_*y*_, and *L*_*z*_, which are 10, 20, 21, and 10, respectively. The normalized length scales of *L*_*y*_ confirms a block ratio of 0.05. To more accurately model the flow in the separation zone behind the square cylinder, an encryption zone of grid is set around the square cylinder. The normalized dimensions of the encrypted regions are denoted as *L*_*1*_, *L*_*2*_, *L*_*3*_ and are chosen to be 4, 10 and 2.5. The boundary condition at the entrance is a velocity inlet where the streamwise wind velocity is given by ${U}_{0}$. The outlet boundary is free flow and the four outer boundaries are symmetric.

Mesh quality affects the stability and accuracy of the simulations. The high-quality meshes also reduce nonphysical calculation errors^22,23^. In our simulations, unstructured meshes are introduced. Most meshes are hexahedral, while very few are tetrahedral. The smallest mesh is attached to the square cylinder surface with a size of 0.0125 m, which is too coarse to resolve the laminar layer. An alternative near-wall approach based on the work of Werner and Wengle^24^, who proposed an analytical integration of the power-law near-wall velocity distribution resulting in the following expressions for the wall shear stress:

1 $$\left| {\tau_{w} } \right| = \left\{ {\begin{array}{*{20}l} {\frac{{2\mu \left| {u_{p} } \right|}}{\Delta z}, \left| {u_{p} } \right| \le \frac{\mu }{2\rho \Delta z}A^{{\frac{2}{1 - B}}} } \hfill \\ {\rho \left[ {\frac{1 - B}{2}A^{{\frac{1 + B}{{1 - B}}}} \left( {\frac{\mu }{\rho \Delta z}} \right)^{B} \left| {u_{p} } \right|} \right]^{{\frac{2}{1 + B}}} , \left| {u_{p} } \right| > \frac{\mu }{2\rho \Delta z}A^{{\frac{2}{1 - B}}} } \hfill \\ \end{array} } \right.,$$

where ${u}_{p}$ is wall-parallel velocity, ${A}=8.3$ and ${B}=1/7$ are the constants, and $\Delta z$ is the near-wall control volume length scale.

The fluctuating velocity components are generated with the method of the spectral synthesizer, which is based on the random flow generation technique originally proposed by Kraichnan^25^ and modified by Smirnov et al.^26^. In this method, fluctuating velocity components are computed by synthesizing a divergence-free velocity-vector field from the summation of Fourier harmonics.

### Governing equations

The filtered Navier–Stokes equations are in the following form:

2 $$\frac{\partial {\overline{u} }_{i}}{\partial {x}_{i}}=0,$$

3 $$\frac{\partial {\overline{u} }_{i}}{\partial t}+{\overline{u} }_{j}\frac{\partial {\overline{u} }_{i}}{\partial {x}_{j}}=-\frac{\partial \rho }{\partial {x}_{i}}+\left[v\left(\frac{\partial {\overline{u} }_{i}}{\partial {x}_{j}}+\frac{\partial {\overline{u} }_{j}}{\partial {x}_{i}}\right)-{\tau }_{ij}\right],$$

The subgrid-scale stress resulting from the filtering operation are unknown, and require modeling. The compressible form of the subgrid stress tensor is defined as ${\tau }_{ij}=\rho \widetilde{{u}_{i}{u}_{j}}-\rho {\widetilde{u}}_{i}{\widetilde{u}}_{j}$. The most of the subgrid models are based on the eddy viscosity model. The dedicator part of the subgrid-scale stress tensor is modeled using the incompressible form if the Smagorinsky model ${\tau }_{ij}-\left({\tau }_{kk}{\delta }_{ij}\right)/3=-{2\mu }_{t}{\overline{S} }_{ij}$. Where ${\mu }_{t}$ is the subgrid-scale turbulent viscosity. ${\overline{S} }_{ij}$ is the rate-of-strain tensor of the resolved scale defined by ${\overline{S} }_{ij}={\widetilde{u}}_{i,j}+{\widetilde{u}}_{j,i}$. The eddy-viscosity is modeled by ${\mu }_{t}=\rho {L}_{S}^{2}\left|\overline{S }\right|$, where ${L}_{S}$ is the mixing length for subgrid scales and $\left|\overline{S }\right|={(2{\overline{S} }_{ij}{\overline{S} }_{ji})}^{0.5}$. The mixing length is defined by ${L}_{S}=min(kd, {C}_{S}, \Delta )$, where $k$ is the von Karman constant, $d$ is the distance to the closest wall, ${C}_{S}$ is the Smagorinsky constant, and $\Delta$ is the local grid scale. A ${C}_{S}$ value of 0.1 has been found to yield the best resits for a widely range of flows.

### LES solving procedure

An incompressible 3D finite volume code is introduced to solve the filtered Navier–Stokes equations. Firstly, the momentum equation (Eq. 3) needs to be discretized. The gradient, pressure and momentum are discretized with least square cell-based scheme, second order accuracy, and bounded central differencing scheme. The transit formation is discretized with a bounded second-order implicit time integration. The solution method is the PISO algorithm, which moves the repeated computation of the momentum equation inside the solution phase of the pressure correction equation. The dimensionless time step is 0.02. The number of iterations is limited to 20 per time step, while the true number of iterations is about 3–5 per time step. The time-dependent data is averaged over 3 time steps, with a physical simulation time of 20 s or 1200 in dimensionless time. The convergence criterion is set to 0.001 because higher convergence criterion of 0.0001 showed no significant changes in the results whereas the number of iterations increases approximately two times^27^.

The filtered Navier–Stokes equations are integrated over a time interval, and over the control volume. Thus the momentum equation (Eq. 3) is transformed into the general matrix form for numerical solution as shown in Eq. (4) below, where the M matrix is the coefficient obtained after discretizing the equation using the finite volume method, and the coefficient matrix is known:

4 $$MU=-\nabla p,$$

The next step is to decompose the coefficient matrix *M* into diagonal and off-diagonal matrices, and Eq. (4) becomes the following equation Eq. (5), where *A* is the diagonal matrix and *H* is the off-diagonal matrix.

5 $$AU-H=-\nabla p,$$

where $H$ is derived explicitly from the non-diagonal matrix term and the velocity term of the previous iteration step, which is part of the source term of the pressure Poisson equation.

Then we invert the decomposed equation to get the explicit velocity equation:

6 $$U={A}^{-1}H-{A}^{-1}\nabla p,$$

Then we can get the pressure Poisson equation by adding Eq. (6) to the continuity equation:

7 $$\nabla \cdot \left({A}^{-1}\nabla p\right)=\nabla \cdot \left({A}^{-1}H\right),$$

The solution procedure of the PISO algorithm is summarized as follows.

1. the momentum equation is solved by the given initial pressure or the pressure of the previous iteration step, but the obtained velocity variable does not necessarily satisfy the continuity equation;
2. the pressure is obtained by solving the Eq. (7);
3. the velocity is modified by the obtained pressure to satisfy the continuity equation;
4. if the velocity does not satisfy the momentum equation, update the Eq. (5) and repeat the cycle.

## Results

### Grid dependency test

**Figure 2:**
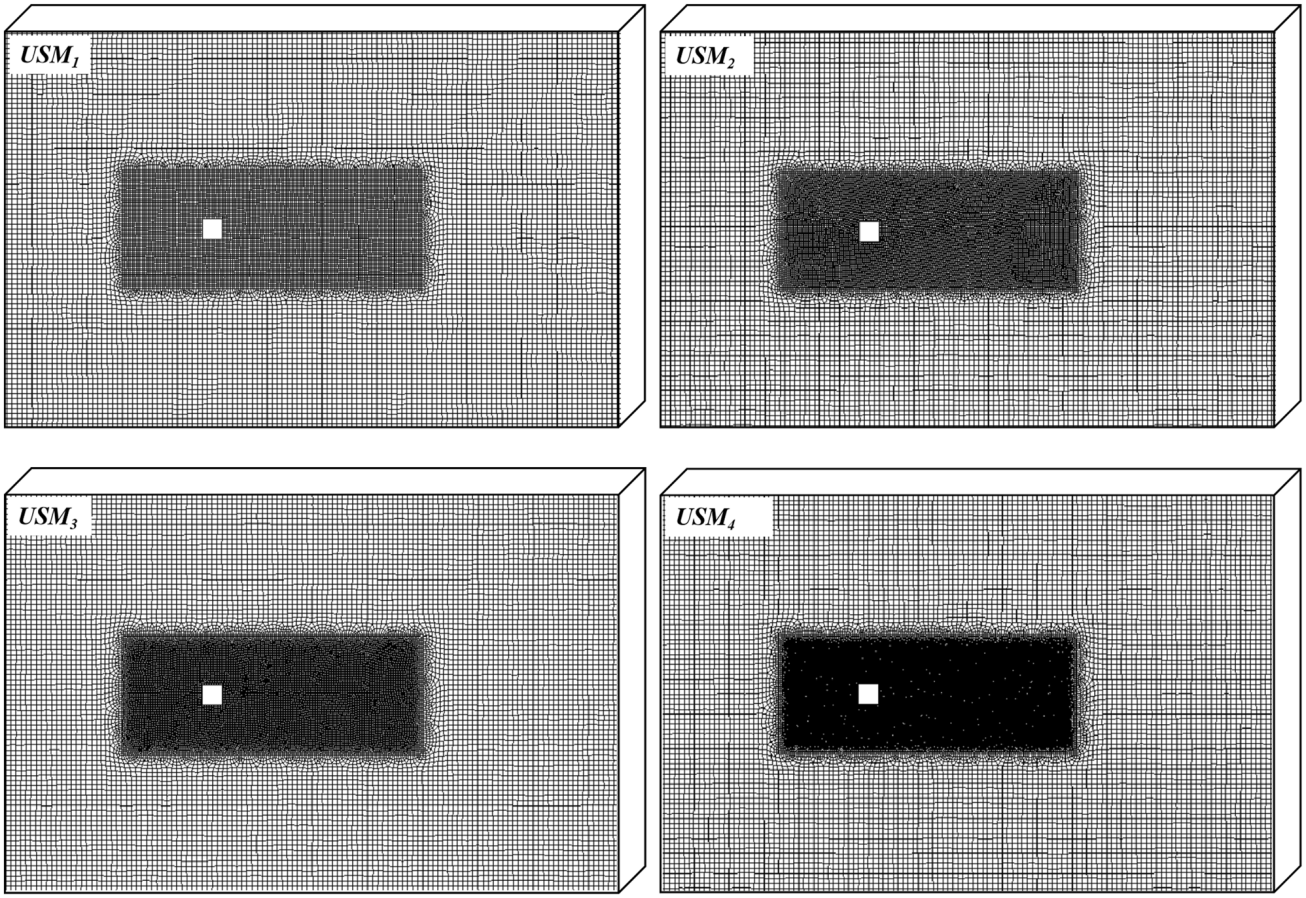
Four mesh resolutions with grid number $7.3\times {10}^{5}$, $9.4\times {10}^{5}$, $12.2\times {10}^{5}$ and $18.2\times {10}^{5}$, respectively.

**Figure 3:**
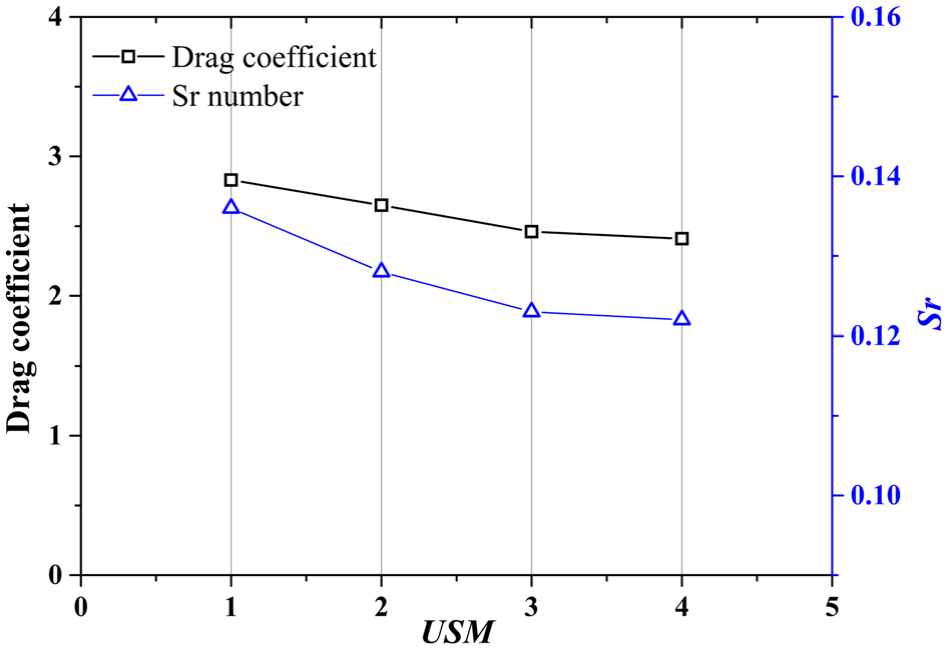
Effect of different mesh resolutions on drag coefficient and Strouhal number of an isolated square cylinder.

In this study, four mesh resolutions, denoted by *USM*_1_ to *USM*_4_, are employed to test the effect of grid on results. Figure 2 shows the gradually thinning grid in the encryption zone. The grid sizes in the encryption zone are $1/10\it{D}$, $1/12.5\it{D}$, $1/16\it{D}$ and $1/20\it{D}$, respectively. The grid sizes in the outer zone of the computation domain are $1/4\it{D}$ in all mesh schemes. The grid size in z direction are $1/4\it{D}$ for all mesh schemes. The total grid number of the four cases are $7.3\times {10}^{5}$, $9.4\times {10}^{5}$, $12.2\times {10}^{5}$ and $18.2\times {10}^{5}$. A comparison between the obtained results by different grids is performed in Fig. 3. By increasing the grid number from *USM*_3_ to *USM*_4_, the deviation of drag coefficient and Strouhal number are less than 5%. As a result, *USM*_3_ is a suitable resolution scheme to provide grid independence.

**Figure 4:**
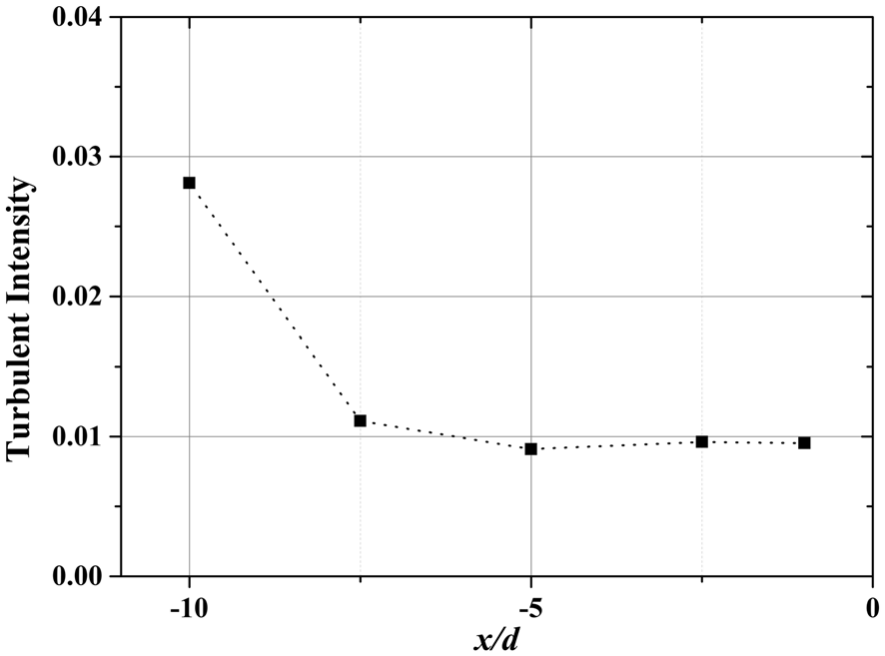
Turbulent intensity along the streamwise direction. ${x}/{d}=-10$ indicates the inlet boundary and ${x}/{d}=-0.5$ indicates the upwind surface of the square cylinder.

The turbulent intensity of *USM*_3_ defined by ${\upsigma }_{u}/\overline{u}$ is presented in Fig. 4, where ${\sigma }_{u}$ and $\overline{u}$ are the standard deviation and averaged velocity in x direction. The turbulent intensity is 0.028 at the entrance of the computation domain, while it decreases along x direction due to the turbulent kinetic energy is dispersed in the other two directions. The turbulent intensity eventually stabilized at 0.01.

### Simulation validation

**Figure 5:**
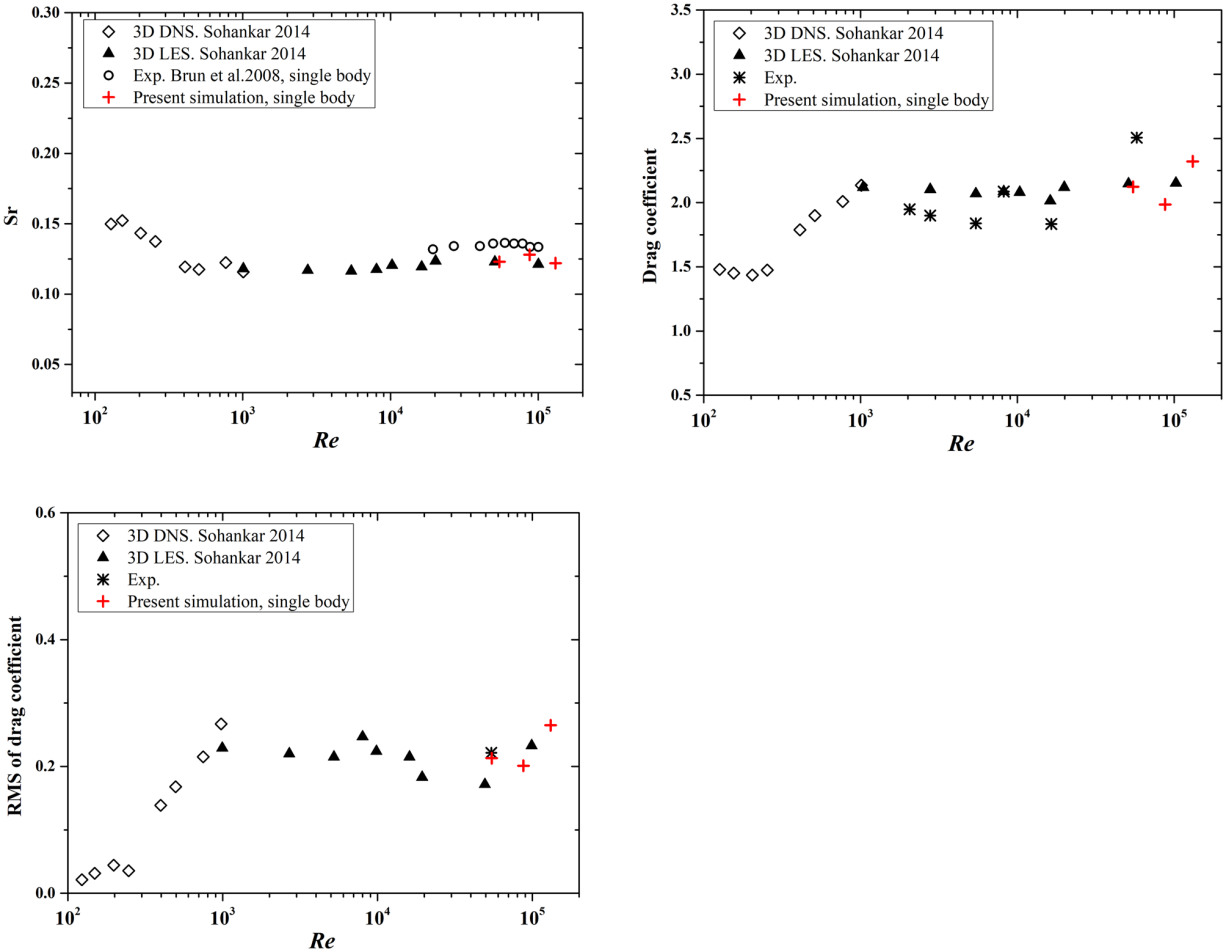
Strouhal number (*Sr*), drag coefficient and RMS drag coefficient with respect to Reynold number (*Re*). The 3D DNS, 3D LES simulation results and experimental results are presented in Sohankar^27^.

To validate the accuracy of the current numerical simulation results, we compare the Strouhal number, time-averaged drag coefficient and RMS of drag coefficient with simulation and experiment results presented in Sohankar^27^. The incoming wind velocities are 5 m/s, 8 m/s, and 12 m/s, corresponding to the Reynolds numbers $5.46\times {10}^{4}$, $8.74\times {10}^{4}$ and $1.31\times {10}^{5}$, respectively. As shown in Fig. 5, the results of this numerical simulation are close to the 3D Les and experimental outputs, which demonstrates the accuracy of the numerical simulation strategy. The simulation setup can be applied to more similar examples.

### Transverse wind load of a square cylinder under periodic fluctuating flow

The transverse wind velocity fluctuates as a sinusoidal function to simulate cross-wind fluctuations. The amplitude of the sine function is 2 (m/s), and the frequency is denoted by ${f}_{v}$. The overall vortex-excited forces (VEF) acting on the square model are the face integral of pressure in the transverse direction. As vortex shedding is synchronous, the VEF is a time-varying variable, denoted by ${F}_{i}(t)$. When the vibrational frequency is close to the natural frequency of the structure, vortex-induced vibrations occur and the structure fails. Therefore, it is necessary to study the dependence of the ${F}_{i}$ vibration frequency on the cross-wind fluctuation frequency of the inlet wind velocity.

**Figure 6:**
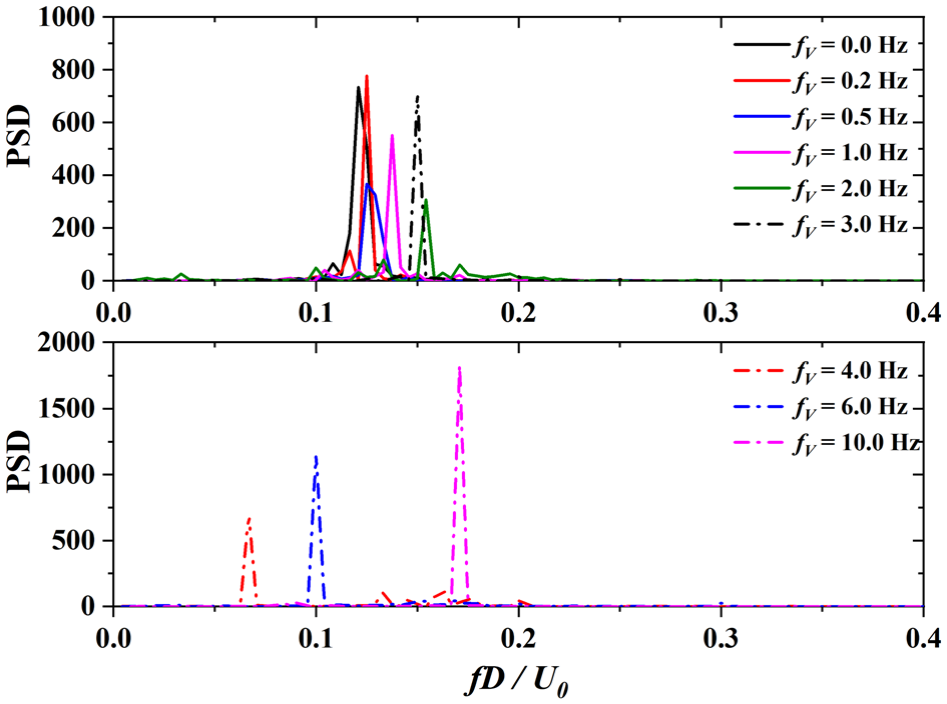
PSD of vortex-excited force acting on the square cylinder for various fluctuation frequencies transversely generated by a sine function at the inlet boundary.

**Figure 7:**
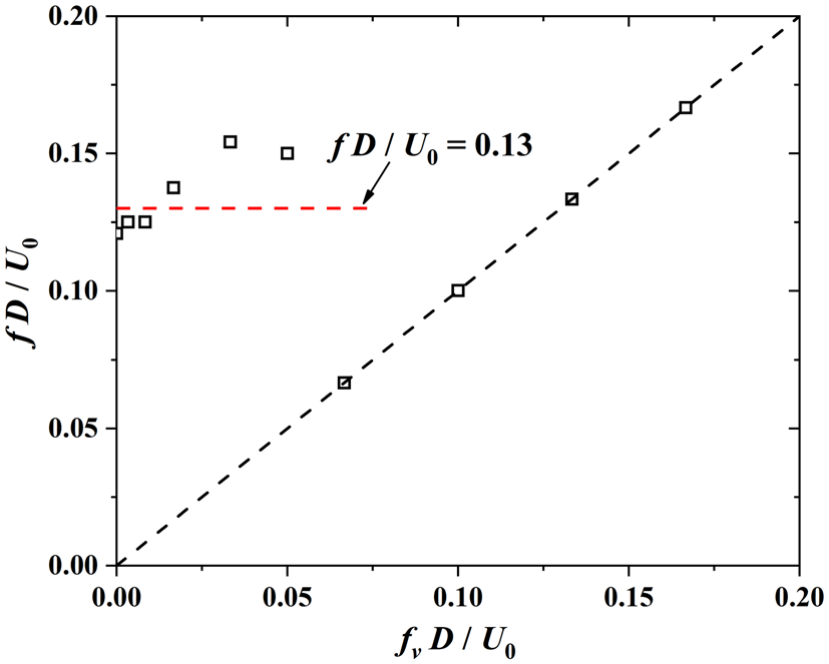
The vibration frequency of the vortex-excited force (*f*) with various cross-wind frequencies at the inlet boundary (${f}_{V}$).

Figure 6 presents the power spectrum density (PSD) of ${F}_{i}(t)$ transformed with FFT. The frequencies are normalized via ${fD}/{{U}}_{0}$. In the cases with ${f}_{V}$ = 0.2 Hz, 0.5 Hz, and 1 Hz, the normalized vibration frequencies of ${F}_{i}$ are 0.124, 0.125, and 0.137, which are close to the *Sr* number 0.121 of ${f}_{V}$ = 0 Hz. As presented in Fig. 7, the normalized vibration frequency $fD/{U}_{0}$ fluctuates around the Strouhal number in the range of ${f}_{V}D/{U}_{0}\le 0.05$, indicating that the low-frequency cross-wind fluctuation has little effect on the transverse vibration of ${F}_{i}$. However, the vibration frequency of ${F}_{i}$ present linear increase with ${f}_{V}D/{U}_{0}>0.05$ Hz, indicating that the cross-wind fluctuation excites the vibration of ${F}_{i}$ significantly.

### VEF under the disturbance from the upwind barriers

**Figure 8:**
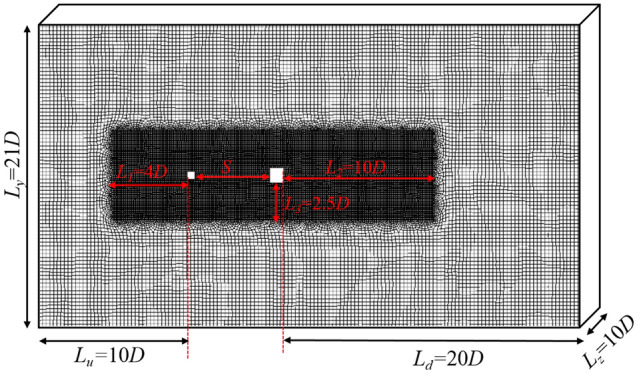
Computational domain of square cylinders with a barrier upwind.

As landmarks of the cities, high-rise buildings are primarily located downtown. No fluctuating wind field in nature follows the sine function strictly. However, the complex urban underlying surface can lead to the periodic fluctuating near-surface wind field, which may cause VIV of high-rise buildings^4^. To verify whether the frequency effect of cross-wind vibration exists on the building behind a barrier, we conducted a simplified simulation. Figure 8 presents the computation of tandem square cylinders. An upwind barrier generates the transverse wind fluctuation at the square cylinder, while five intervals (*S*) between the barrier and the square cylinder are used. The sizes of the barrier are denoted by *H.* All computational cases of tandem square cylinders are listed in Table 1.

**Table 1:** Computational cases and details of two tandem square cylinder.

Case No	*H/D*	*S/D*	z grid No	x–y grid No	$\frac{{L}_{y}}{D}$	$\frac{{L}_{u}}{D}$	$\frac{{L}_{d}}{D}$	$\frac{{L}_{1}}{D}$	$\frac{{L}_{2}}{D}$	$\frac{{L}_{3}}{D}$
1–1	0.5	2	40	39,778	21	10	20	5	10	2.5
1–2		5		45,409						
1–3		8		51,138						
1–4		10		55,003						
1–5		15		64,225						
2–1	1	2	40	40,931	21	10	20	5	10	2.5
2–2		5		46,464						
2–3		8		52,034						
2–4		10		56,001						
2–5		15		65,042						
3–1	2.5	2	40	88,046	31	10	40	5	10	5
3–2		5		101,726						
3–3		8		113,581						
3–4		10		121,176						
3–5		15		138,592						

**Figure 9:**
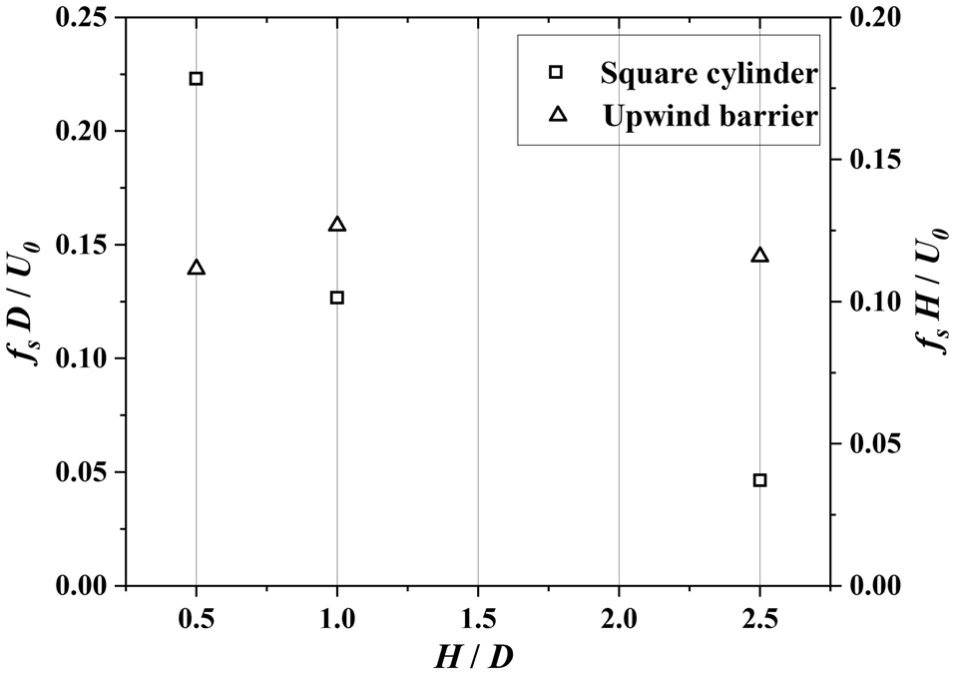
Transverse wind fluctuation generated by upwind barrier with various sizes and the corresponding Strouhal number, *S* = 2 *D.*

The frequency of the transverse wind fluctuations at the downstream square cylinder depends on the size of the barrier. As presented in Fig. 9, due to the disturbance from the upwind barrier, the normalized frequency of transverse wind acting on the square cylinder decrease with the size of the upwind barrier. However, the Strouhal number of the upwind barrier is around 0.124, which is close to that present in Sohankar^27^.

**Figure 10:**
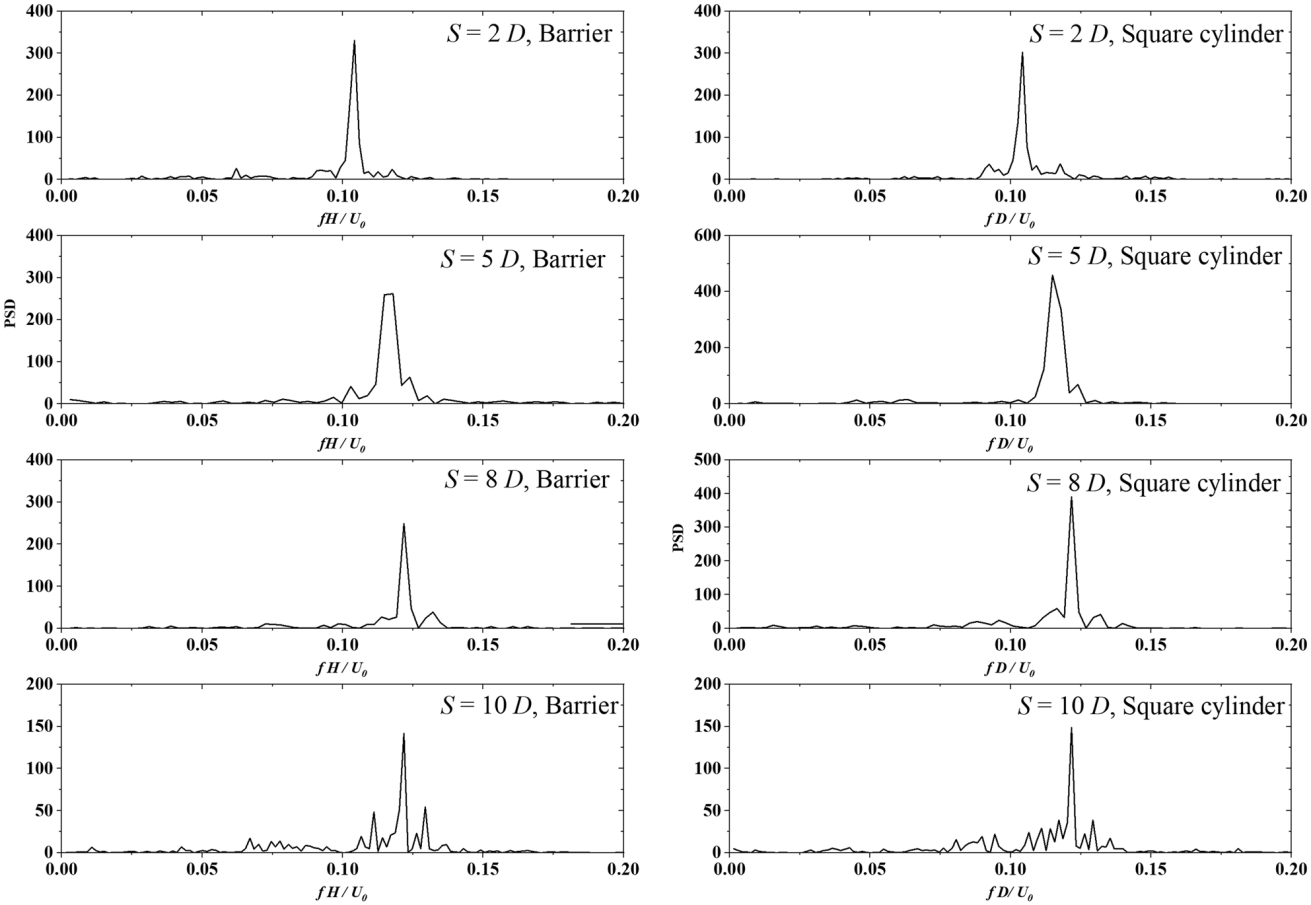
${F}_{i}$ power spectrum density of the upwind barrier and the downwind square cylinder with *H* = *D*.

Figure 10 shows the ${F}_{i}$ power spectral density of the upwind barrier and the downwind square cylinder for Cases 2–1 and 2–3 listed in Table 1. Single peaks are detected at the upwind barrier and the downwind square cylinder. With *S* = 2 *D*, the normalized peak frequency of the upwind barrier and the square cylinder is 0.104, which is lower than the Strouhal number of an isolated square cylinder. The interaction between the closely arranged barrier and the square cylinder decreases the vibration frequency by 20%. As S increases to 5 *D*, the normalized peak frequency of the upwind barrier and the square cylinder increases to 0.121, which is close to the expected value of the Strouhal number. In addition, the peak value of the square barrier is enhanced, indicating superposition vibrations.

**Figure 11:**
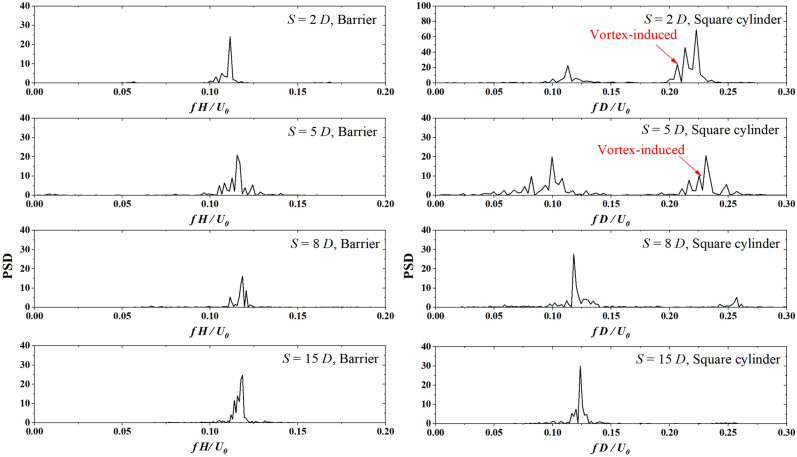
${F}_{i}$ power spectrum density of the upwind barrier and the downwind square cylinder with *H* = 0.5 *D*.

Figure 11 presents the ${F}_{i}$ power spectrum density of the upwind barrier and the downwind square cylinder for Cases 1–1 to 1–3. Narrow intervals of *S* = 2 *D* and 5 *D* lead to a decrease in the Strouhal number of the barrier, indicating the influence from the downwind square cylinder. As the interval S increases to 8 *D*, the Strouhal number of the barrier remains stable at 0.12 and no longer changes with *S*. The square cylinder exhibits two peaks with *S* = 2 *D* and 5 *D*. The first peak is caused by the cross-wind fluctuation induced by the upwind barrier. The second peak can be detected around 0.25. As the interval S reaches 8 *D*, the second peak vanishes, indicating that the interaction from the barrier vanishes.

**Figure 12:**
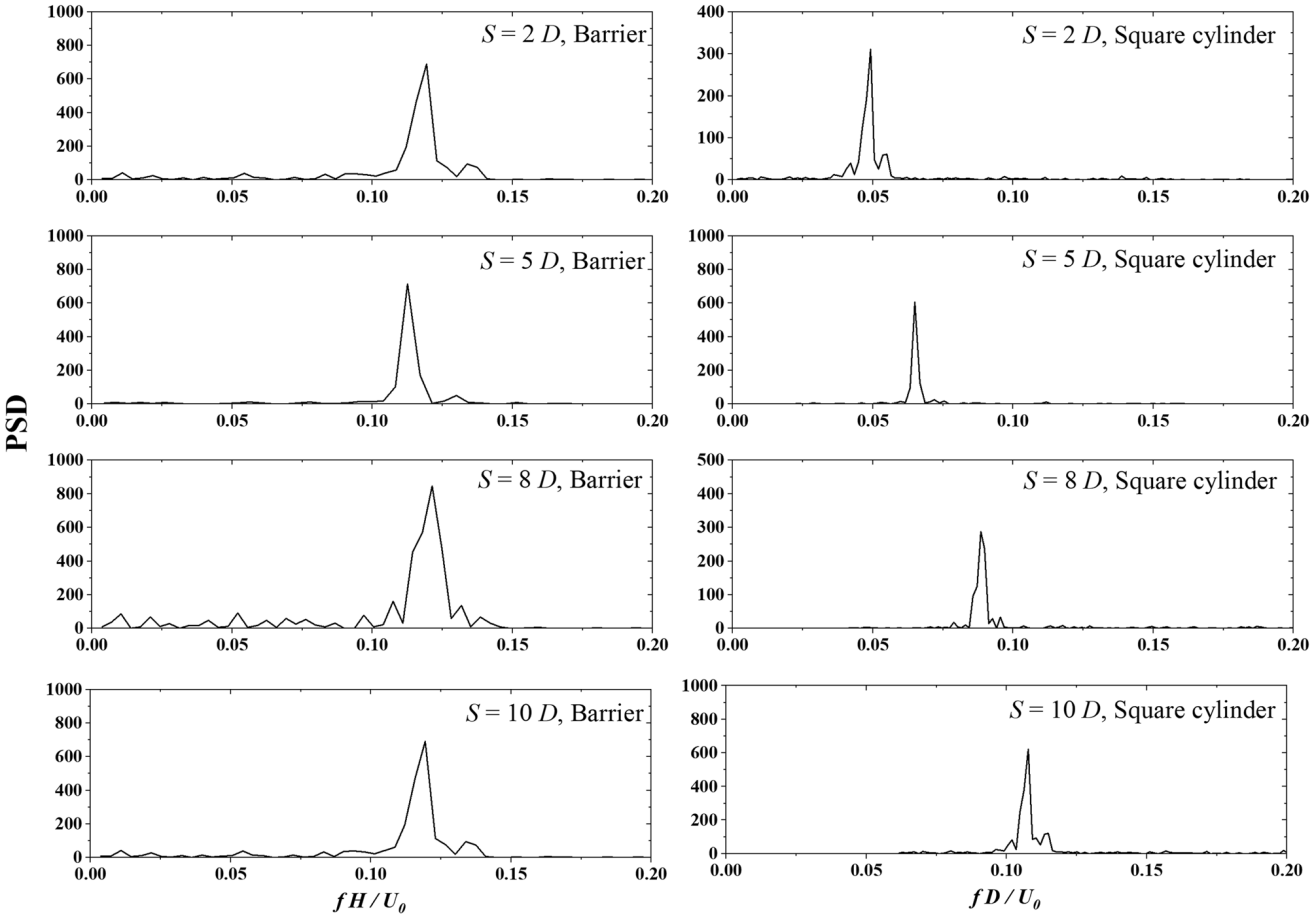
${F}_{i}$ power spectrum density of the upwind barrier and the downwind square cylinder with *H* = 2.5 *D*.

Figure 12 presents the ${F}_{i}$ power spectral density at the upwind barrier and the square cylinder for Cases 3–1 to 3–3*.* The normalized peak frequency of the upwind barrier increases with *S*, and becomes stable at *S* = 8 *D*, indicating damping from the downwind influence of the square cylinder. However, as D increases from 2 to 10 *D*, the normalized peak frequency of the square cylinder rises from 0.052 to 0.113. The upwind barrier is larger than the square cylinder in this case. The narrow spacing decreases the reference wind velocity at the square cylinder, leading to a decrease in the normalized peak frequency.

By comparing Figs. 11 and 12, we confirm that the frequency effects of the transverse wind fluctuations discussed in Section “Transverse wind load of a square cylinder under periodic fluctuating flow” are still present on the square cylinder in the wake region of a barrier. For barriers of sizes 0.5 *D* and 2.5 *D*, the normalized shedding frequencies in the wake region are about 0.223 and 0.046, respectively. According to the results in Fig. 6, the former can excite the square cylinder at the same frequency, while the latter cannot be detected in the vortex-induced force signal of the square cylinder because its value is below 0.05. The incoming flow fluctuating at a lower frequency leads to vortices with larger spatial scales. Larger vortices tend to bypass the square cylinder, causing most of the turbulent kinetic energy not to act on the square cylinder. In contrast, when the incoming flow fluctuates at higher frequencies, the spatial wavelength of the vortex is shorter. The vortices can barely bypass the square cylinder, leading to a significant excitation effect of the short wavelength waves received by the square cylinder.

## Conclusion

In this study, we investigated the corsswind drag force on a square cylinder affected by transverse wind fluctuation. A given sine function at the inlet boundary and an upwind barrier generated the transverse wind fluctuation. The wind flow is simulated via LES with Smagorinsky sub-grid turbulence model. The transverse drag force of the square cylinder is analyzed. Based on the simulation results, we draw the following conclusions:

- With transverse wind fluctuation generated by a sine function, PSD of vortex-excited force shows that for ${f}_{V}D/{U}_{0}$ = 0.003, 0.008, and 0.017, the normalized vibration frequencies of the square cylinder are 0.125, 0.125, and 0.137, which are close to the expected Sr number 0.13. As ${f}_{V}D/{U}_{0} >0.05$, the cross-wind vibration frequency controls the vibration frequency of the vortex-excited force.
- With transverse wind fluctuation generated by an upwind barrier with the same size of target square cylinder, results show that the interaction between the closely arranged barrier and the square cylinder decreases the natural vibration frequency by 20%. This interaction disappears after *S* reaches 8 *D*.
- With transverse wind fluctuation generated by an upwind barrier half size of the target square cylinder, results show that the square cylinder exhibits two peaks with *S* = 2 *D* and 5 *D.* The second peak is caused by the cross-wind fluctuation induced by the upwind barrier. The first peak can be detected between 0.113 and 0.125 due to the natural vibration frequency of the square cylinder. As the interval *S* reaches 8 *D*, the second peak vanishes, indicating the interaction from the barrier disappears.
- With transverse wind fluctuation generated by an upwind barrier 2.5-time size of the target square cylinder, results show that with *S* increasing from 2 to 10 *D*, the normalized peak frequencies of the square barrier increase from 0.052 to 0.113. The significant decrease in normalized peak frequency is due to the reduction in the reference wind velocity at the square cylinder.

## Data Availability

The datasets used and/or analysed during the current study available from the corresponding author on reasonable request.
